# *N*-Cyclohexyl-*N*-methylbenzene­sulfonamide

**DOI:** 10.1107/S1600536809041762

**Published:** 2009-10-28

**Authors:** Zeeshan Haider, Islam Ullah Khan, Muhammad Nadeem Arshad, Muhammad Shafiq, Caoyuan Niu

**Affiliations:** a Materials Chemistry Laboratory, Department of Chemistry, GC University, Lahore 54000, Pakistan; b College of Sciences, Henan Agricultural University, Zhengzhou 450002, People’s Republic of China

## Abstract

The title compound, C_13_H_19_NO_2_S, was synthesized by the reaction of *N*-cyclo­hexyl­amine­benzene­sulfonamide and methyl iodide. The crystal packing is stabilized by weak inter­molecular C—H⋯O hydrogen bonds.

## Related literature

Compounds containing cyclo­hexyl­amine have been reported to be activators of dopamine receptors in the central nervous system, see: Hacksell *et al.* (1981 ▶). For related structures, see: Arshad *et al.* (2008 ▶, 2009 ▶).
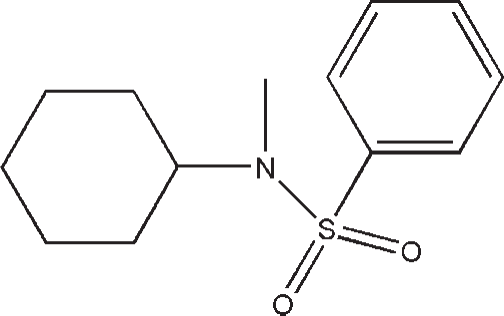


## Experimental

### 

#### Crystal data

C_13_H_19_NO_2_S
*M* *_r_* = 253.35
Monoclinic, 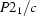

*a* = 9.2729 (5) Å
*b* = 12.1182 (7) Å
*c* = 12.5801 (7) Å
β = 109.103 (2)°
*V* = 1335.79 (13) Å^3^
*Z* = 4
Mo *K*α radiation
μ = 0.23 mm^−1^
*T* = 296 K
0.28 × 0.12 × 0.09 mm

#### Data collection

Bruker APEXII CCD detector diffractometer
Absorption correction: multi-scan (*SADABS*; Bruker, 2005 ▶) *T*_min_ = 0.938, *T*_max_ = 0.979
12741 measured reflections
2489 independent reflections
1864 reflections with *I* > 2σ(*I*)
*R*_int_ = 0.030

#### Refinement

*R*[*F* ^2^ > 2σ(*F* ^2^)] = 0.038
*wR*(*F* ^2^) = 0.113
*S* = 1.08
2489 reflections
155 parameters
H-atom parameters constrained
Δρ_max_ = 0.16 e Å^−3^
Δρ_min_ = −0.25 e Å^−3^

### 

Data collection: *APEX2* (Bruker, 2005 ▶); cell refinement: *SAINT* (Bruker, 2005 ▶); data reduction: *SAINT*; program(s) used to solve structure: *SHELXS97* (Sheldrick, 2008 ▶); program(s) used to refine structure: *SHELXL97* (Sheldrick, 2008 ▶); molecular graphics: *SHELXL97* and *DIAMOND* (Brandenburg, 2005 ▶); software used to prepare material for publication: *SHELXL97*.

## Supplementary Material

Crystal structure: contains datablocks I, global. DOI: 10.1107/S1600536809041762/bt5092sup1.cif

Structure factors: contains datablocks I. DOI: 10.1107/S1600536809041762/bt5092Isup2.hkl

Additional supplementary materials:  crystallographic information; 3D view; checkCIF report

## Figures and Tables

**Table 1:** Hydrogen-bond geometry (Å, °)

*D*—H⋯*A*	*D*—H	H⋯*A*	*D*⋯*A*	*D*—H⋯*A*
C2—H2⋯O2^i^	0.93	2.52	3.268 (3)	137

Symmetry code: (i) 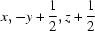.

**Fig. 1.**
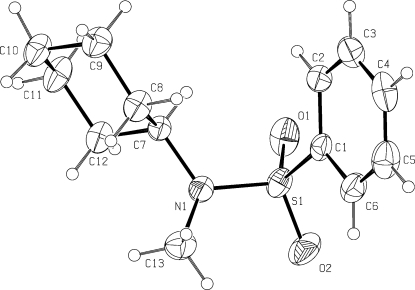
The molecular structure of the title compound showing the atom-labelling scheme. Displacement ellipsoids are drawn at the 30% probability level.

**Fig. 2.**
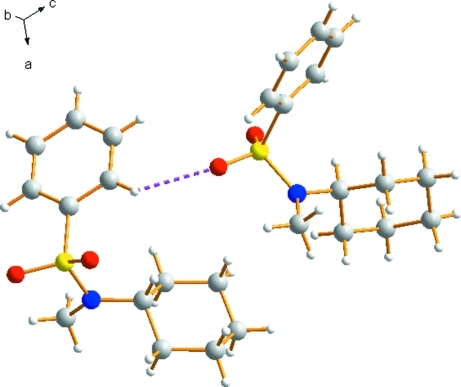
Diagram showing the intermolecular hydrogen bonds (indicated by pink dashed lines).

## supplementary crystallographic information

### Comment

Sulfonamide  compounds have gained much importance due to their therapeutic applications. The compound containing cyclohexylamine has been reported to be an activator of dopamine receptors in the *CNS* (Hacksell *et al.*, 1981). The title compound is a sulfonamide derivative of cyclohexylamine in continuation to our previous work (Arshad *et al.*, 2008; Arshad *et al.*, 2009).

The molecular structure of the title compound (I) is shown in Fig. 1.  The mean plane of the benzene ring and that of the four essentially planar C atoms (C8, C9, C11, C12. Maximum deviation, 0.0132 Å) of the chair-form cyclohexyl ring have a dihedral angle of 24.26 (9)°. Furthermore, there are intermolecular C—H···O hydrogen bonds between the aromatic H atom (H2) and one sulfonamide O atom (O2^i^, symmetric code: see table 1) of neighboring molecules that contribute to the three-dimensional packing  (Fig. 2).

### Experimental

Sodium hydride (0.88 mmol) was taken in a round bottom flask and washed with n-hexane so as to remove the mineral oil dispersant. A solution of *N*-cyclohexylamine benzene sulfonamide (0.43 mmol) in 5 ml of N,*N* dimethyl formamide was added. The mixture was stirred for half an hour at room temperature. Then,  methyl iodide (0.86 mmol) was added and stirring was continued for about 3 hrs until the complete consumption of sulfonamide. The reaction was monitored by TLC. After the completion of the reaction the contents were transferred into the distilled water ice. The product  precipitated and was separated by filtration and recrystallized from methanol. The melting point of the product was observed to be 353 K uncorrected.

### Refinement

All   H atoms were placed in calculated positions and refined using a riding model [C—H = 0.93 Å and *U*_iso_(H) = 1.2*U*_eq_(C) for aromatic H atoms; C—H = 0.98 Å and *U*_iso_(H) = 1.2*U*_eq_(C) for tertiary CH; C—H = 0.97 Å and *U*_iso_(H) = 1.2*U*_eq_(C) for CH_2_; C—H = 0.96 Å and *U*_iso_(H) = 1.5*U*_eq_(C) for the methyl H atoms]. The final difference Fourier map had a highest peak at 0.71 Å from atom C1 and a deepest hole at 0.71 A Å from atom S1, but were otherwise featureless.

### Crystal data

**Table:**

C_13_H_19_NO_2_S	*F*(000) = 544
*M**_r_* = 253.35	*D*_x_ = 1.260 Mg m^−^^3^
Monoclinic, *P*2_1_/*c*	Mo *K*α radiation, λ = 0.71073 Å
Hall symbol: -P 2ybc	Cell parameters from 3617 reflections
*a* = 9.2729 (5) Å	θ = 2.3–25.5°
*b* = 12.1182 (7) Å	µ = 0.23 mm^−^^1^
*c* = 12.5801 (7) Å	*T* = 296 K
β = 109.103 (2)°	Block, colourless
*V* = 1335.79 (13) Å^3^	0.28 × 0.12 × 0.09 mm
*Z* = 4	

### Data collection

**Table:**

Bruker APEXII CCD detector diffractometer	2489 independent reflections
Radiation source: fine-focus sealed tube	1864 reflections with *I* > 2σ(*I*)
graphite	*R*_int_ = 0.030
phi and ω scans	θ_max_ = 25.5°, θ_min_ = 2.3°
Absorption correction: multi-scan (*SADABS*; Bruker, 2005)	*h* = −10→11
*T*_min_ = 0.938, *T*_max_ = 0.979	*k* = −14→14
12741 measured reflections	*l* = −13→15

### Refinement

**Table:**

Refinement on *F*^2^	Primary atom site location: structure-invariant direct methods
Least-squares matrix: full	Secondary atom site location: difference Fourier map
*R*[*F*^2^ > 2σ(*F*^2^)] = 0.038	Hydrogen site location: inferred from neighbouring sites
*wR*(*F*^2^) = 0.113	H-atom parameters constrained
*S* = 1.08	*w* = 1/[σ^2^(*F*_o_^2^) + (0.0559*P*)^2^ + 0.2086*P*] where *P* = (*F*_o_^2^ + 2*F*_c_^2^)/3
2489 reflections	(Δ/σ)_max_ < 0.001
155 parameters	Δρ_max_ = 0.16 e Å^−^^3^
0 restraints	Δρ_min_ = −0.25 e Å^−^^3^

### Special details

**Table:**

Geometry. All e.s.d.'s (except the e.s.d. in the dihedral angle between two l.s. planes) are estimated using the full covariance matrix. The cell e.s.d.'s are taken into account individually in the estimation of e.s.d.'s in distances, angles and torsion angles; correlations between e.s.d.'s in cell parameters are only used when they are defined by crystal symmetry. An approximate (isotropic) treatment of cell e.s.d.'s is used for estimating e.s.d.'s involving l.s. planes.
Refinement. Refinement of *F*^2^ against ALL reflections. The weighted *R*-factor *wR* and goodness of fit *S* are based on *F*^2^, conventional *R*-factors *R* are based on *F*, with *F* set to zero for negative *F*^2^. The threshold expression of *F*^2^ > σ(*F*^2^) is used only for calculating *R*-factors(gt) *etc*. and is not relevant to the choice of reflections for refinement. *R*-factors based on *F*^2^ are statistically about twice as large as those based on *F*, and *R*- factors based on ALL data will be even larger.

### Fractional atomic coordinates and isotropic or equivalent isotropic displacement parameters (Å^2^)

**Table:**

	*x*	*y*	*z*	*U*_iso_*/*U*_eq_	
C1	0.67239 (19)	0.36402 (15)	0.21562 (15)	0.0443 (5)	
C2	0.6576 (2)	0.33212 (18)	0.31729 (17)	0.0556 (5)	
H2	0.7103	0.2710	0.3556	0.067*	
C3	0.5644 (2)	0.3918 (2)	0.3605 (2)	0.0694 (7)	
H3	0.5552	0.3717	0.4294	0.083*	
C4	0.4849 (3)	0.4804 (2)	0.3038 (2)	0.0738 (7)	
H4	0.4200	0.5192	0.3332	0.089*	
C5	0.5005 (3)	0.51236 (19)	0.2034 (2)	0.0732 (7)	
H5	0.4467	0.5731	0.1652	0.088*	
C6	0.5951 (2)	0.45494 (17)	0.15921 (18)	0.0568 (5)	
H6	0.6070	0.4772	0.0918	0.068*	
C7	1.0474 (2)	0.29629 (15)	0.33830 (14)	0.0424 (4)	
H7	0.9836	0.2401	0.3570	0.051*	
C8	1.0716 (2)	0.38752 (18)	0.42469 (16)	0.0568 (5)	
H8A	0.9739	0.4194	0.4203	0.068*	
H8B	1.1338	0.4452	0.4084	0.068*	
C9	1.1495 (2)	0.3434 (2)	0.54232 (16)	0.0632 (6)	
H9A	1.1678	0.4037	0.5958	0.076*	
H9B	1.0829	0.2907	0.5610	0.076*	
C10	1.2988 (2)	0.2885 (2)	0.55192 (17)	0.0651 (6)	
H10A	1.3416	0.2563	0.6262	0.078*	
H10B	1.3701	0.3434	0.5430	0.078*	
C11	1.2786 (3)	0.1995 (2)	0.46425 (18)	0.0679 (7)	
H11A	1.2203	0.1392	0.4805	0.082*	
H11B	1.3780	0.1710	0.4682	0.082*	
C12	1.1976 (2)	0.24159 (18)	0.34615 (16)	0.0546 (5)	
H12A	1.1787	0.1805	0.2936	0.065*	
H12B	1.2625	0.2943	0.3255	0.065*	
C13	1.0145 (3)	0.4353 (2)	0.18252 (19)	0.0719 (7)	
H13A	0.9857	0.4965	0.2197	0.108*	
H13B	0.9659	0.4421	0.1027	0.108*	
H13C	1.1233	0.4350	0.1996	0.108*	
N1	0.96659 (17)	0.33232 (13)	0.22141 (13)	0.0485 (4)	
O1	0.78750 (17)	0.17730 (12)	0.18902 (14)	0.0710 (5)	
O2	0.75416 (18)	0.32058 (15)	0.04381 (11)	0.0769 (5)	
S1	0.79429 (6)	0.29004 (4)	0.15969 (4)	0.0523 (2)	

### Atomic displacement parameters (Å^2^)

**Table:**

	*U*^11^	*U*^22^	*U*^33^	*U*^12^	*U*^13^	*U*^23^
C1	0.0369 (9)	0.0421 (11)	0.0442 (10)	−0.0061 (8)	0.0002 (7)	−0.0009 (8)
C2	0.0458 (11)	0.0601 (14)	0.0563 (12)	−0.0031 (10)	0.0104 (9)	0.0119 (10)
C3	0.0505 (12)	0.0910 (19)	0.0690 (15)	−0.0087 (13)	0.0227 (11)	−0.0028 (13)
C4	0.0447 (12)	0.0776 (18)	0.098 (2)	−0.0046 (12)	0.0213 (13)	−0.0252 (15)
C5	0.0569 (14)	0.0515 (14)	0.098 (2)	0.0069 (11)	0.0078 (13)	0.0027 (13)
C6	0.0513 (12)	0.0506 (13)	0.0579 (12)	0.0002 (10)	0.0033 (10)	0.0078 (10)
C7	0.0397 (10)	0.0442 (11)	0.0410 (10)	−0.0014 (8)	0.0101 (8)	0.0003 (8)
C8	0.0558 (12)	0.0591 (13)	0.0535 (12)	0.0128 (10)	0.0151 (9)	−0.0100 (10)
C9	0.0655 (14)	0.0758 (16)	0.0468 (12)	0.0075 (11)	0.0163 (10)	−0.0124 (10)
C10	0.0573 (13)	0.0805 (17)	0.0477 (12)	0.0090 (11)	0.0039 (10)	−0.0097 (11)
C11	0.0618 (14)	0.0700 (16)	0.0583 (13)	0.0212 (11)	0.0009 (10)	−0.0093 (11)
C12	0.0519 (12)	0.0567 (13)	0.0507 (12)	0.0091 (9)	0.0108 (9)	−0.0131 (9)
C13	0.0738 (15)	0.0693 (16)	0.0675 (15)	−0.0071 (12)	0.0162 (12)	0.0176 (12)
N1	0.0454 (9)	0.0507 (10)	0.0462 (9)	−0.0010 (7)	0.0108 (7)	0.0024 (7)
O1	0.0658 (10)	0.0425 (9)	0.0905 (11)	−0.0052 (7)	0.0064 (8)	−0.0131 (8)
O2	0.0797 (11)	0.1027 (13)	0.0379 (8)	0.0063 (9)	0.0050 (7)	−0.0119 (8)
S1	0.0513 (3)	0.0512 (3)	0.0449 (3)	−0.0004 (2)	0.0027 (2)	−0.0090 (2)

### Geometric parameters (Å, °)

**Table:**

C1—C6	1.378 (3)	C9—C10	1.505 (3)
C1—C2	1.385 (3)	C9—H9A	0.9700
C1—S1	1.760 (2)	C9—H9B	0.9700
C2—C3	1.369 (3)	C10—C11	1.509 (3)
C2—H2	0.9300	C10—H10A	0.9700
C3—C4	1.364 (3)	C10—H10B	0.9700
C3—H3	0.9300	C11—C12	1.517 (3)
C4—C5	1.374 (4)	C11—H11A	0.9700
C4—H4	0.9300	C11—H11B	0.9700
C5—C6	1.372 (3)	C12—H12A	0.9700
C5—H5	0.9300	C12—H12B	0.9700
C6—H6	0.9300	C13—N1	1.462 (3)
C7—N1	1.481 (2)	C13—H13A	0.9600
C7—C8	1.515 (3)	C13—H13B	0.9600
C7—C12	1.516 (3)	C13—H13C	0.9600
C7—H7	0.9800	N1—S1	1.6144 (16)
C8—C9	1.516 (3)	O1—S1	1.4218 (16)
C8—H8A	0.9700	O2—S1	1.4302 (15)
C8—H8B	0.9700		
			
C6—C1—C2	120.5 (2)	H9A—C9—H9B	108.0
C6—C1—S1	119.68 (16)	C9—C10—C11	111.51 (18)
C2—C1—S1	119.84 (15)	C9—C10—H10A	109.3
C3—C2—C1	119.1 (2)	C11—C10—H10A	109.3
C3—C2—H2	120.5	C9—C10—H10B	109.3
C1—C2—H2	120.5	C11—C10—H10B	109.3
C4—C3—C2	120.8 (2)	H10A—C10—H10B	108.0
C4—C3—H3	119.6	C10—C11—C12	112.27 (18)
C2—C3—H3	119.6	C10—C11—H11A	109.2
C3—C4—C5	120.1 (2)	C12—C11—H11A	109.1
C3—C4—H4	120.0	C10—C11—H11B	109.1
C5—C4—H4	120.0	C12—C11—H11B	109.1
C6—C5—C4	120.2 (2)	H11A—C11—H11B	107.9
C6—C5—H5	119.9	C11—C12—C7	111.14 (17)
C4—C5—H5	119.9	C11—C12—H12A	109.4
C5—C6—C1	119.4 (2)	C7—C12—H12A	109.4
C5—C6—H6	120.3	C11—C12—H12B	109.4
C1—C6—H6	120.3	C7—C12—H12B	109.4
N1—C7—C8	113.90 (15)	H12A—C12—H12B	108.0
N1—C7—C12	110.43 (15)	N1—C13—H13A	109.5
C8—C7—C12	110.80 (15)	N1—C13—H13B	109.5
N1—C7—H7	107.1	H13A—C13—H13B	109.5
C8—C7—H7	107.1	N1—C13—H13C	109.5
C12—C7—H7	107.1	H13A—C13—H13C	109.5
C7—C8—C9	110.76 (17)	H13B—C13—H13C	109.5
C7—C8—H8A	109.5	C13—N1—C7	118.27 (15)
C9—C8—H8A	109.5	C13—N1—S1	118.12 (13)
C7—C8—H8B	109.5	C7—N1—S1	118.92 (12)
C9—C8—H8B	109.5	O1—S1—O2	119.63 (10)
H8A—C8—H8B	108.1	O1—S1—N1	107.52 (9)
C10—C9—C8	111.50 (17)	O2—S1—N1	107.13 (10)
C10—C9—H9A	109.3	O1—S1—C1	107.27 (10)
C8—C9—H9A	109.3	O2—S1—C1	106.79 (9)
C10—C9—H9B	109.3	N1—S1—C1	108.06 (8)
C8—C9—H9B	109.3		

### Hydrogen-bond geometry (Å, °)

**Table:**

*D*—H···*A*	*D*—H	H···*A*	*D*···*A*	*D*—H···*A*
C2—H2···O2^i^	0.93	2.52	3.268 (3)	137

Symmetry codes: (i) *x*, −*y*+1/2, *z*+1/2.
